# The utility of procalcitonin for the patients with infected pancreatic necrotic and pancreatic abscess

**DOI:** 10.1186/2197-425X-3-S1-A113

**Published:** 2015-10-01

**Authors:** T Adachi, Y Kishihara, H Okano, H Honzawa, M Hirayama, H Higashi, H Yasuda, Y Minami, S Hara, N Harada, A Katsumi, S Suzaki

**Affiliations:** Emergency and Critical Care Medicine, Japan Red Cross Musashino Hospital, Tokyo, Japan

## Introduction

There are many complications of severe acute pancreatitis (SAP), but infected pancreatic necrosis and pancreatic abscess are very serious and relate to the mortality and morbidity. Recently there have been many studies about the utility of procalcitonin (PCT) as a factor of prediction for infective complications in SAP.

## Objectives

To clarify the utility of PCT as a factor of prediction for infected pancreatic necrosis and pancreatic abscess.

## Methods

In a single center of the Advanced Emergency Medical Center in Japan, the 25 patients diagnosed as SAP in accordance with the criteria of Japanese Ministry of Health, Labour and Welfare. We measured their PCT once a day during the first week after hospitalization and three times a week after first week during they were in the hospital. The fine needle aspiration (FNA) was administered, when we suspect the existence of infective complication during the course of SAP. The definition of existence of infective complication is to be bacteriologically-positive. Comparative review was conducted on the PCT level of both groups. Review 1 is to compare the maximum PCT level of with and without infected complication, both were recorded within 1 week after hospitalization. Review 2 is to compare the maximum PCT level of with infected complication recorded during 1 week before proved infection and that of without recorded within 1 week after hospitalization.

## Results

The 19 of the 25 patients of SAP ware included because 6 patients of them had been excluded due to passing away within 1 week. Of the remaining 19 patients, 9 represented infective complication (infected group) and 10 were none (non-infected group). All the infected complication developed at least 1 week after their hospitalization. The infected group's median age, APACHE II score, and SOFA score were, respectively, 73 years old (interquartile range (IQR) 55 to 80), 18(IQR 13 to 22), and 7(IQR 3 to 10), those of the non-infected group were 49 years old (IQR 43 to 73), 12(IQR 9 to 20), and 4(IQR 3 to 8), which show no significant differences. Review 1: The infected group's maximum PCT level was higher, but have largely varied and shown no significant difference (2.3µg/dl vs.0.8µg/dl : p = 0.09). Review 2: The maximum PCT level of the infected group was found to have increased with statistical significance as compared to that of the non-infected group (0.7µg/dl vs. 0.2µg/dl : p = 0.05). Assuming the PCT cut off value being 0.5µg/dl, the sensitivity and specificity of PCT during the infective complication were 0.55 and 0.9, respectively.

## Conclusions

While the elevated PCT during course management of SAP will possibly give an indication of the infective complication, its clinical application requires further collection of the clinical data as it is no more than just slightly higher than the upper limit of normal level according our results of this study.

